# Supplementary material for: BRD4 Inhibition as a Strategy to Prolong the Response to Standard of Care in Estrogen Receptor-Positive Breast Cancer
Source: Cancers (Basel). 2023 Aug 11;15(16):4066. doi: 10.3390/cancers15164066 (PMC10452571; doi:10.3390/cancers15164066)
Supplement: Supplementary file 1 [file cancers-15-04066-s001.zip › cancers-2506097-supplementary.pdf]

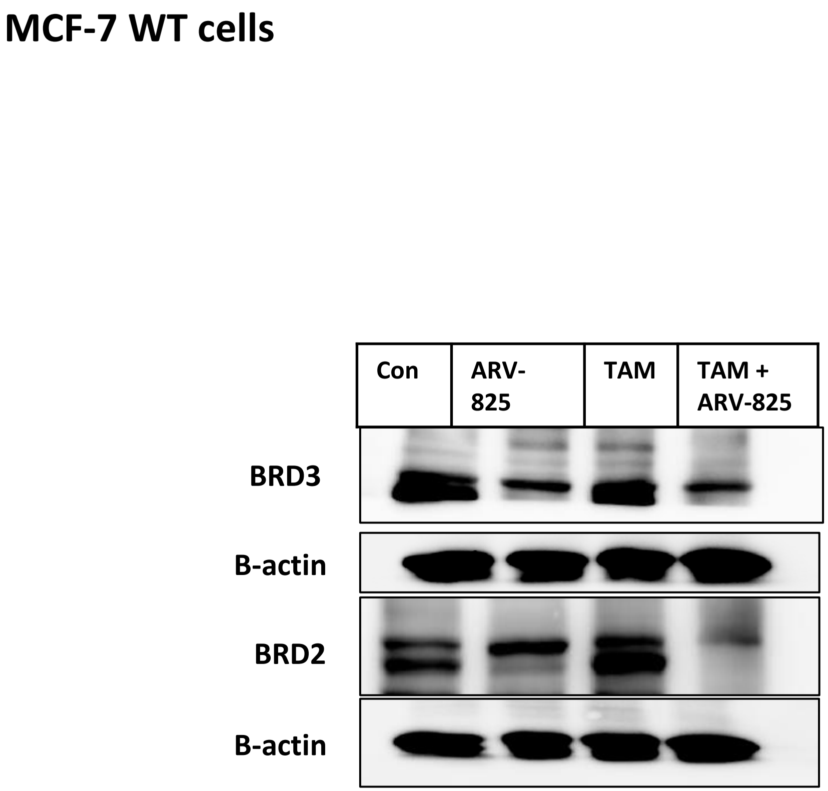

Supplemental Figure S1. ARV-825 suppresses levels of BRD2 and BRD3. Western blotting for BRD2 and BRD3 after 4 days of ARV-825 treatment. All images are representative blots from at least two/three independent experiments.

MCF-7 WT cells

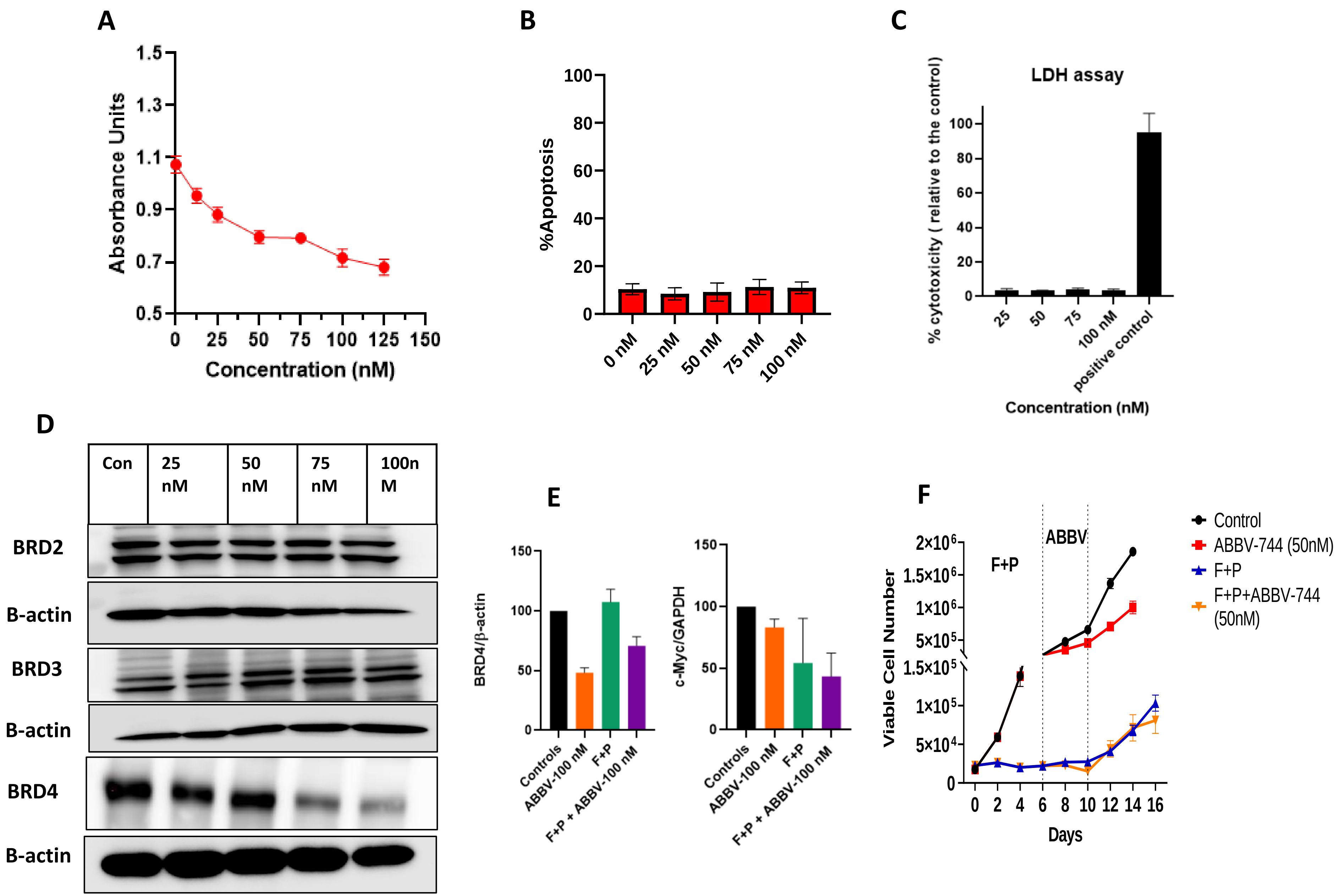

Supplemental Figure S2. ABBV-744 induces a growth inhibitory response in MCF-7 cells without apoptosis induction. (A) Cells were treated with ABBV-744 (12.5, 25, 50, 75, 100 and 125 nM) for 4 days; a dose response curve was determined using the MTS viability assay. (B) Cells were treated with ABBV-744 (25, 50, 75, and 100 nM) for 4 days. Apoptosis was evaluated by flow cytometry using an Annexin V-FTIC Apoptosis Detection Kit at day 4 of the indicated treatments of ABBV-744. (C) Cytotoxicity was measured using the LDH assay at day 4 of the indicated treatments of ABBV-744**.** (D) Western blotting for BRD2, BRD3 and BRD4 at day 4 of ABBV-744 treatments. (E) Quantification of BRD4 and c-Myc western blots in Figure 3E using Image J software. (F) MCF-7 cells were treated with fulvestrant (100 nM) plus palbociclib (1 μM) for 6 days followed by ABBV-744 (50 nM) addition starting from day 6 to 10. Cell viability was monitored over a period of 16 days by trypan blue exclusion. All images are representative fields or blots from at least two/three independent experiments.

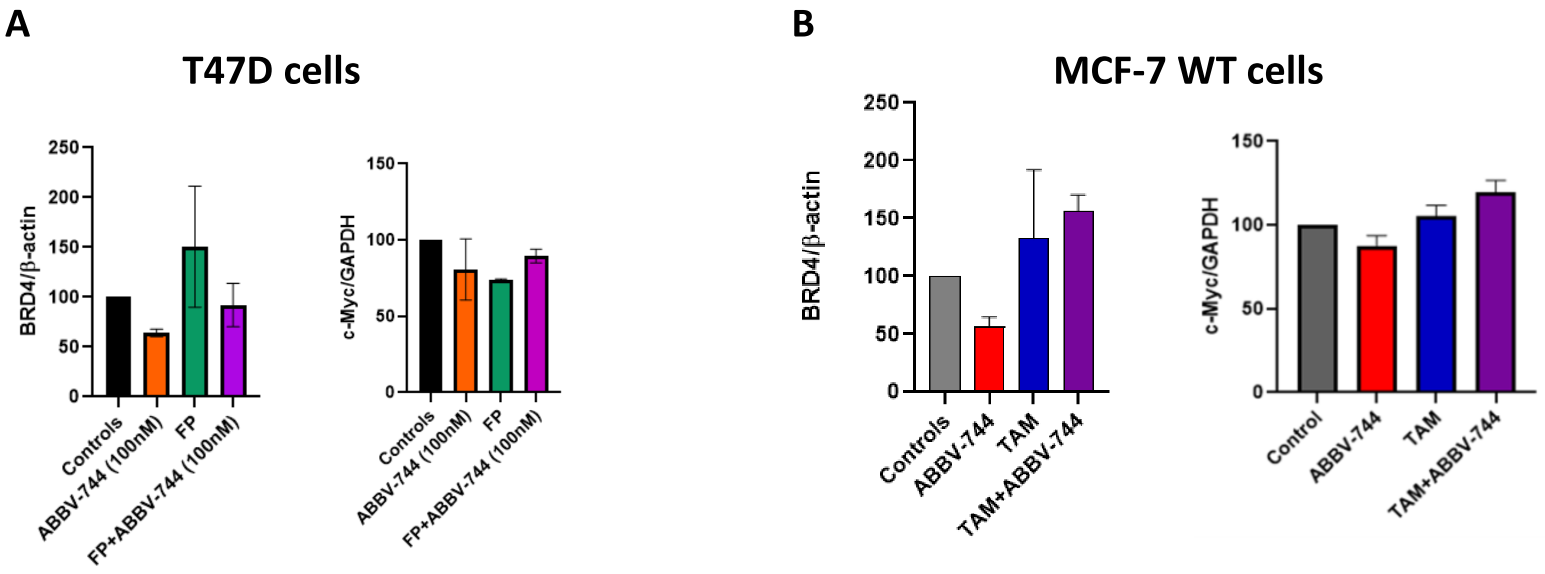

Supplemental Figure S3. ABBV-744-mediated growth inhibition depends on BRD4 suppression when combined with fulvestrant plus palbociclib. (A and B) Quantification of BRD4 and c-Myc western blots in Figure 5F and 6C using Image J software, respectively. All images are representative fields or blots from at least two/three independent experiments.

Original blots and quantification

Original blots for Figure 1E

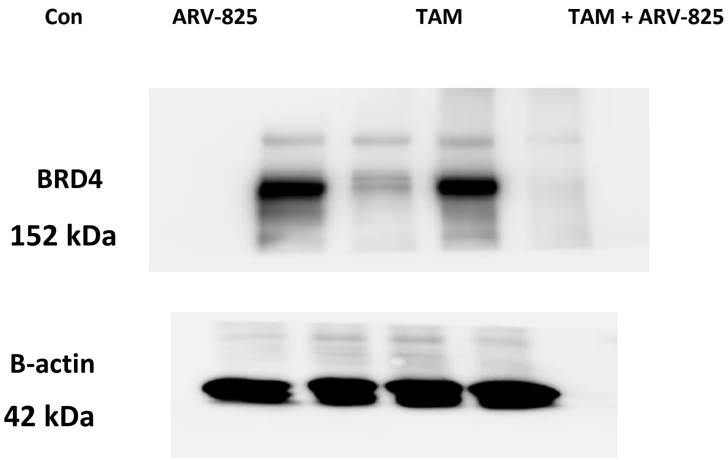

Normalized BRD4:B-actin ratio	1	0.40	0.85	0.19

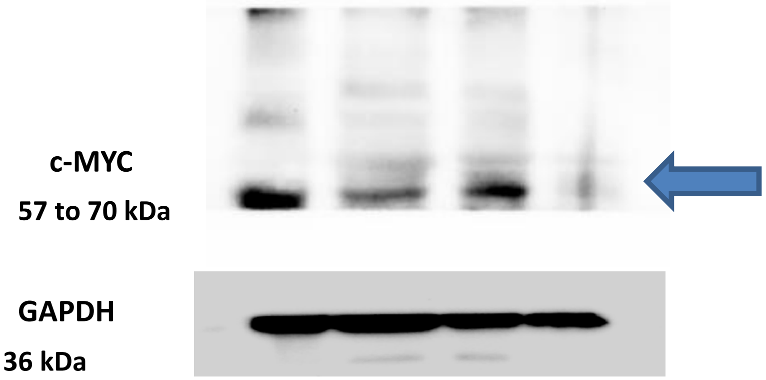

Normalized C-Myc:GAPDH ratio	1	0.66	1.06	0.35

Original blots for Figure 2E

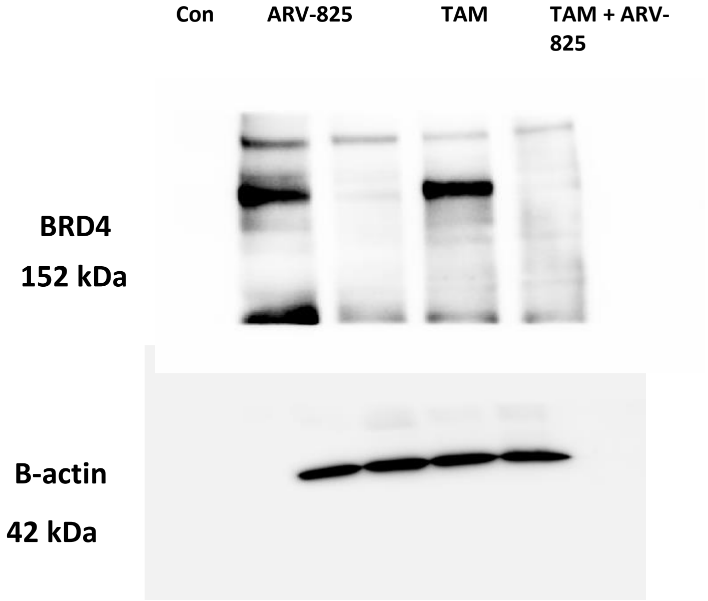

Normalized BRD4:B-actin ratio	1	0.08	0.78	0.11

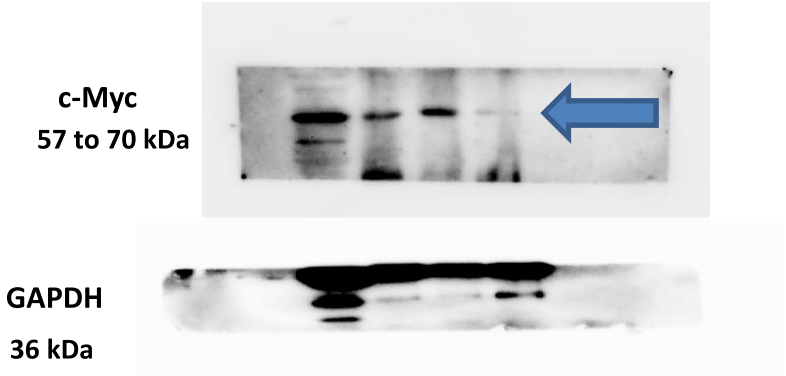

Normalized C-Myc:GAPDH ratio	1	0.72	0.84	0.40

Original blots for Figure 2A

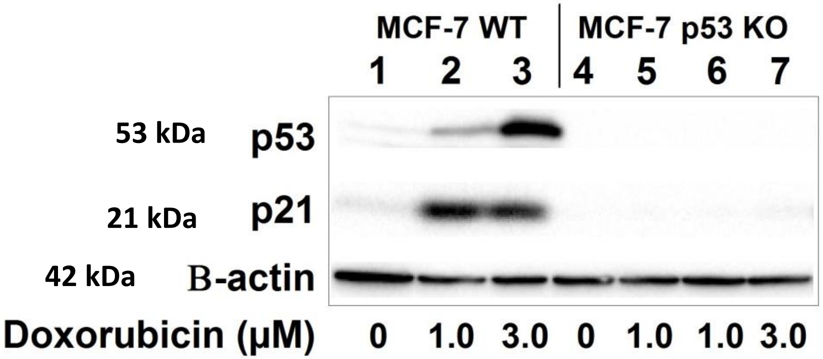

Normalized p53:B-actin ratio	1	8.41	26.28	0.68	0.31	0.26	0.31

Normalized p21:B-actin ratio	1	14.85	11.24	0.78	0.33	0.29	0.65

Original blots for Figure 3E

Con	ABBV-744 (100nM)	FP	FP+ ABBV-744 (100nM)

BRD-4

152 kDa

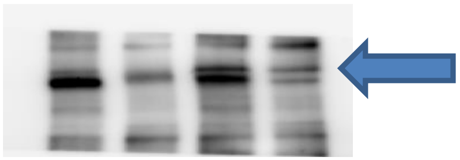

B-actin

42 kDa

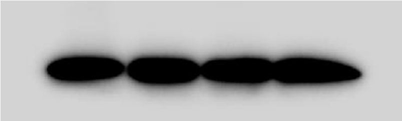

Normalized BRD4:B-actin ratio	1	0.48	1.07	0.70

c-Myc

57 to 70 kDa

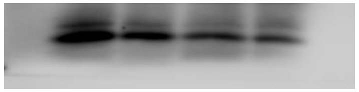

GAPDH

36 kDa

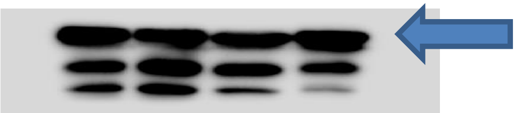

Normalized c-Myc:GAPDH ratio	1	0.83	0.54	0.43

53 kDa p53

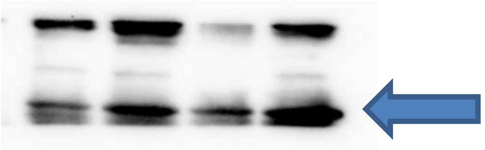

36 kDa GAPDH

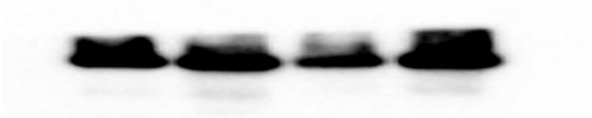

Normalized p53:GAPDH ratio	1	1.20	1.09	1.48

Original blots for Figure 3F

Control BRD4 IP p53 IP

BRD-4

152 kDa

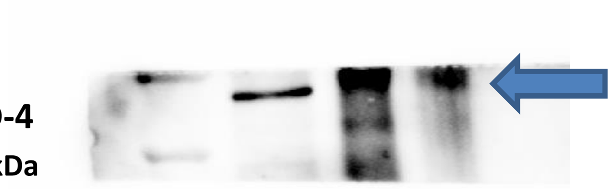

Normalized intensity	1	2.05	1.66

53 kDa p53

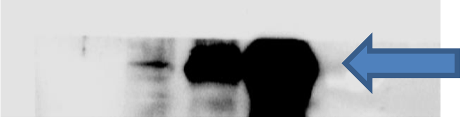

Normalized intensity	1	2.48	3.34

Original blots for Figure 4C

Con	ABBV-744 (100nM)	FP	FP+ ABBV-744 (100nM)

BRD4
152 kDa

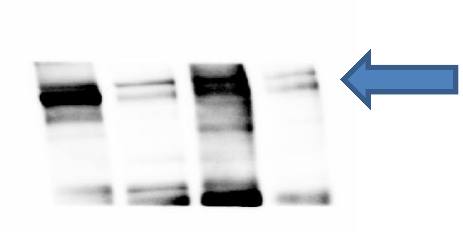

42 kDa B-actin

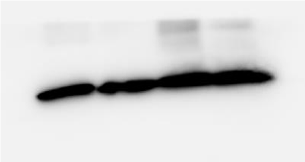

Normalized BRD4:B-actin ratio	1	0.14	0.79	0.11

c-Myc
57 to 70 kDa

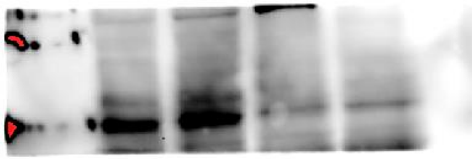

36 kDa GAPDH

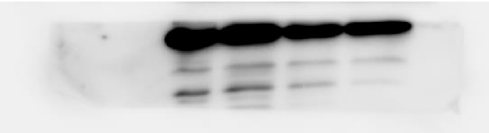

Normalized c-Myc:GAPDH ratio	1	1.06	0.69	0.64

Original blots for Figure 5F

Con	ABBV-744	FP	FP + ABBV-744

BRD-4
152 kDa

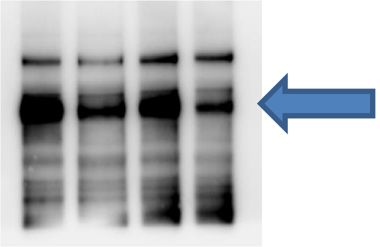

B-actin
42 kDa

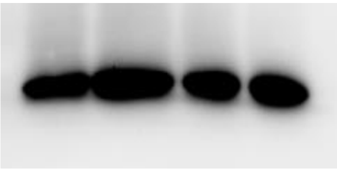

Normalized BRD4:B-actin ratio	1	0.64	1.50	0.92

c-Myc
57 to 70 kDa

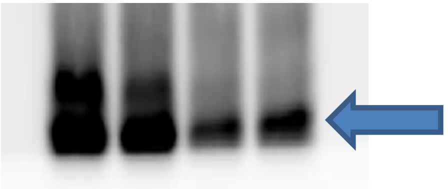

GAPDH
36 kDa

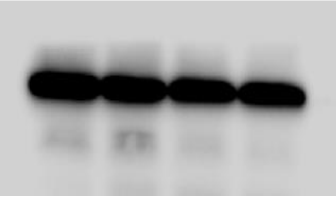

Normalized c-Myc:GAPDH ratio	1	0.80	0.73	0.89

Original blots for Figure 6C

Con	ABBV-744 (100nM)	TAM	TAM + ABBV-744(100nM)

BRD4

152 kDa

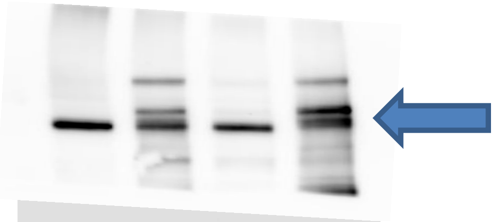

42 kDa B-actin

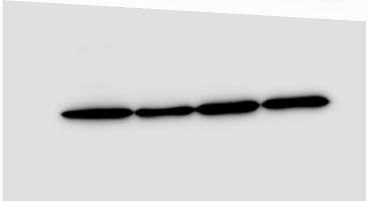

Normalized BRD4:B-actin ratio	1	0.56	1.32	1.56

c-Myc

57 to 70 kDa

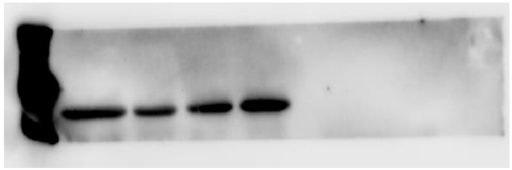

36 kDa GAPDH

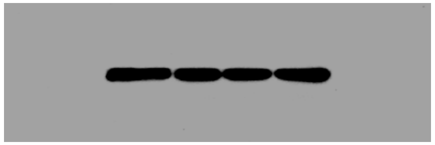

Normalized c-Myc:GAPDH ratio	1	0.87	1.11	1.12

p53

53 kDa

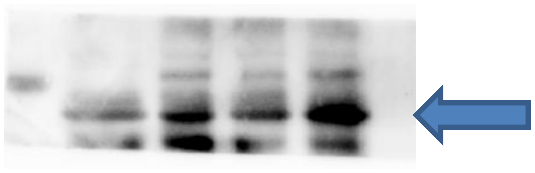

GAPDH

36 kDa

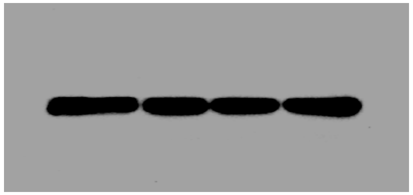

Normalized p53:GAPDH ratio	1	1.49	1.42	2.14

Original blots for Supplementary Figure 1

Con ARV-825 TAM TAM + ARV-825

95-105 kDa BRD3

42 kDa B-actin

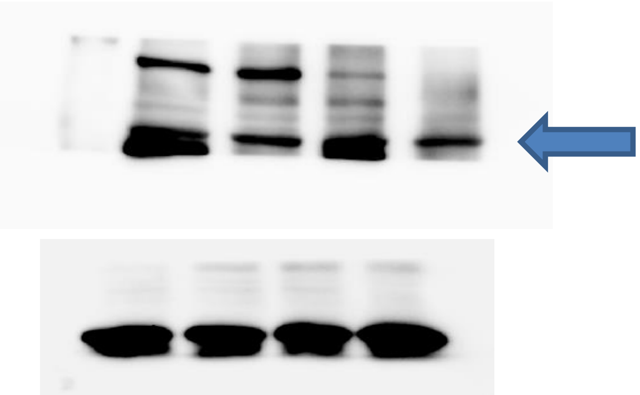

Normalized BRD3:B-actin ratio	1	0.53	0.77	0.51

92 kDa BRD2

42 kDa B-actin

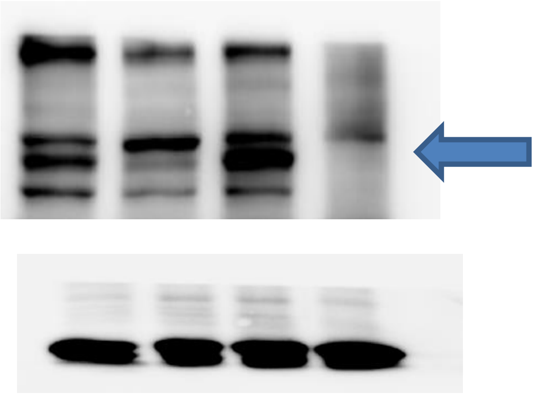

Normalized BRD2:B-actin ratio	1	0.94	1.06	0.47

Original blots for Supplementary Figure 2D

con	25nM	50nM	75nM	100nM

BRD2

92 kDa

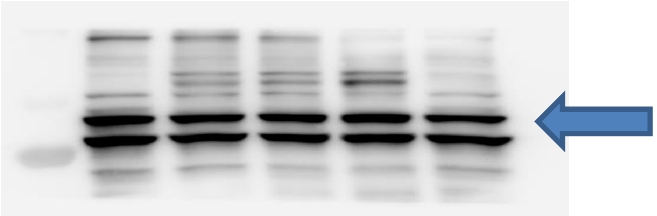

B-actin

42 kDa

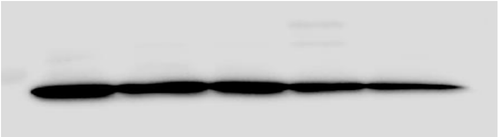

Normalized BRD2:B-actin ratio	1	0.96	0.92	1.09	1.23

BRD3

95-105 kDa

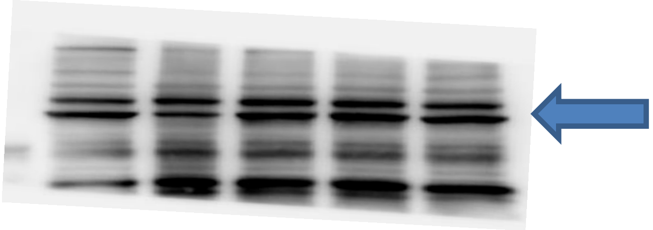

B-actin

42 kDa

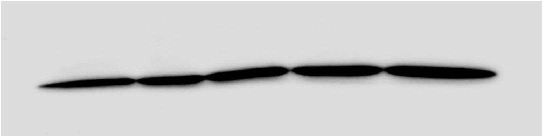

Normalized BRD3:B-actin ratio	1	1.19	1.10	1.30	1.23

BRD4

152 kDa

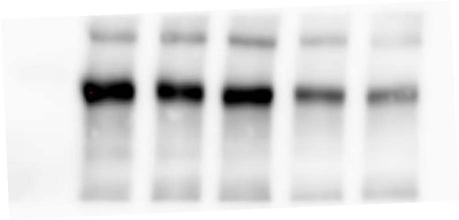

B-actin

42 kDa

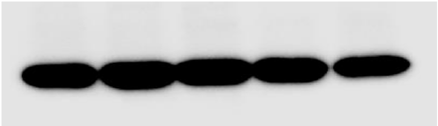

Normalized BRD4:B-actin ratio	1	1.16	0.96	1.14	0.49
